# Why Universities Should Make Misconduct Reports Public

**DOI:** 10.1017/jme.2025.39

**Published:** 2025

**Authors:** Ivan Oransky, Adam Marcus

**Affiliations:** 1 Retraction Watch, United States; 2 New York University, New York, United States; 3 The Transmitter, Simons Foundation, New York, New York, United States; 4 Medscape, Newark, New Jersey, United States

**Keywords:** research misconduct, fraud, investigations, universities

## Abstract

This paper reflects on the availability of a key document in the research integrity landscape: Reports of institutional and university misconduct investigations. It reviews how universities have typically responded to calls for disclosure, offers suggestions to mitigate concerns, and argues that the failure to release such reports creates a critical evidence gap. It closes with a call for disclosure of such reports as a default.

## Introduction

In 2012, the US Office of Research Integrity (ORI) announced it had found Terry Elton, a researcher at The Ohio State University, had committed misconduct by falsifying or fabricating several Western blots in his papers and grant applications.^1^ The case seemed straightforward.

But as the *Columbus Dispatch* reported early in 2013, Ohio State had, in July 2011, following anonymous allegations a year earlier, determined Elton had made some errors in images because of “disorganization,” not “intentional malfeasance.”^2^ The ORI had been unsatisfied with Ohio State’s original investigation, and instructed them to conduct another. That second investigation led to ORI’s finding of misconduct as well as a three-year federal funding ban for Elton.

We know about the earlier finding only because the *Dispatch* filed a public records request and was able to obtain reports and correspondence between ORI and Ohio State. Nor was it the only time during that period a university was ordered to repeat an investigation. It happened again at the University of Florida when an ORI investigator concluded the institution “didn’t do their due diligence” when it found that a researcher had falsified data in only one paper.^3^ After the university hired a new director of research compliance, it found intentional falsifications or fabrications in nine more articles.

As Loikith and Bauchwitz note, “The Percentage of Dismissed Allegations of Biomedical Research Misconduct is Remarkably High.”^4^ Examining ORI data, they found that “almost 90% of biomedical research misconduct allegations continue to be dismissed without receiving an initial inquiry or generating any other specific record or detailed report to ORI.”^5^

Ohio State — whose internal workings are much more available to scrutinize because it is a public institution in a US state with progressive public records laws — has since become far more transparent, following not just the Elton case but that of Carlo Croce that made the front page of the *New York Times* because Croce had escaped findings of misconduct in at least five different investigations.^6^ In 2018, in an unusual move for any institution, the university proactively released a misconduct investigation report about a different researcher and announced the target’s resignation.^7^

The vast majority of misconduct investigation reports, however, remain hidden from view. While summaries from the ORI and the US National Science Foundation’s (NSF’s) Office of Inspector General are useful, they are not very detailed, and in the case of the NSF are carefully anonymized. The annual number of reports from both agencies — generally only totaling in the dozens — also represents a tiny fraction of the number of cases of misconduct. Reports of institutional investigations, even when lacking, serve as signals to the public and other stakeholders of potential problems in academia. Regardless of the outcome of the inquiries, both the number and nature of the resulting reports provide at least a partial indication of what can go wrong in the research endeavor and offer a lower bound for the scope of such issues.

And while public universities like Ohio State are subject to laws pertaining to the disclosure of public records, in our experience, such statutes in many states are not helpful for these types of records even for public universities. Some exempt investigation reports because they are considered personnel records; others require requesters to be residents of the relevant state; and still others consider all investigation reports to be drafts by claiming they are subject to revision until some final — and often malleable — decision by a state or federal agency. Some of these exemptions can be successfully challenged by specialized attorneys, but that step comes at an expense. Universities often use exorbitant charges, based on the costs of legal review of relevant documents, to win their wars of attrition against requesters. And private universities are not subject to public records laws at all, of course.

All of this serves to create an evidence gap in our understanding of effective strategies and tactics to improve research integrity and training. As Armond et al. note, “The lack of publicly available summaries of misconduct investigations makes it difficult to share experiences and evaluate the effectiveness of policies and training programs.”^8^

We believe this gap is bad for transparency, bad for the public, and bad for science. As Redman notes, “Keeping RM [research misconduct] invisible likely is aimed at protecting the authority of science, leaving the assumption in the cases that become public scandals, that science is self-regulating.”^9^ Recent experience suggests that in many cases, science is failing to self-regulate, prioritizing self-interests — on the part of both institutions and individuals — over reform. The existence of schemes such as citation cartels, paper mills, rigged peer review, and other abuses are clear indications many scientists are willing to take steps to game the publishing system. The rapid encroachment of artificial intelligence into the production of journal articles poses perhaps the largest threat yet to the integrity of scientific research. Given recent history, we have no reason to be optimistic about the outcome here. Taken together, all of these factors highlight the paramount importance of transparency in both the conduct of research and in the disclosure of information when such conduct goes astray.

## When reports are available

It doesn’t have to be this way, and some nations have taken steps toward transparency. Laws vary, and making generalizations about the provisions is difficult. However, regulations in certain countries can be considered models, in whole or in part. The United Kingdom’s Concordat, for example, requires universities to provide summary statistics about their investigations, but not reports themselves or even summaries of each investigation.^10^ At least one country, Japan, requires its universities to release reports of misconduct investigations. While these reports are technically anonymized, the context within them — particularly the lists of specific papers affected — generally makes determining the names of the researchers simple. However, universities do not release the sorts of full investigation reports that would be useful to understand how the process unfolded and to identify opportunities for improvement.

The Danish Committees on Scientific Dishonesty is a trio of independent commissions within the Ministry of Research and Information Technology that investigate allegations of misconduct; the Netherlands Board on Research Integrity offers advice to national research institutions on the cases they choose to investigate; and in Belgium two bodies, the Flemish Committee for Scientific Integrity and the Commissions on Research Integrity oversee such inquiries.

Notably, the authors of a recent analysis^11^ initially considered 12 countries but were forced to limit their investigation to three because the rest cited regulations governing confidentiality and data protection or did not provide useful information. While the closed nature of these reports makes further analysis challenging, the study “improves the understanding of how investigations of (alleged) misconduct are handled by the investigating committees in Europe.”^12^ We believe that more such analyses would at the very least allow policymakers to benchmark quality, and from there improve it. Ideally, governments could get involved to waive confounding regulations, with suitable protections for privacy and other concerns, to encourage participation. While the US ORI, following pushback from universities, elected not to loosen confidentiality guidelines for reports following a recent review of its regulations, we hope the agency will address this matter in the future in a way that prioritizes openness.^13^

## Assessing report quality

Some countries, for example Brazil, have little to no consistency in guidance about how to investigate allegations, let alone how to construct useful reports.^14^ In 2018, we proposed a checklist^15^ to, as others have noted, “help standardize investigations into allegations of research misconduct and detrimental research practices.”^16^ The 26 items in the checklist were divided between the general scope of the reports, the makeup and practice of the relevant committee, the evidence they had access to and how they assessed it, and the conclusions they drew.^17^

In 2018, Grey et al. reviewed three institutional reports of investigations their sleuthing efforts had prompted in Japan.^18^ They found “[o]nly 4/78 individual checklist items were addressed adequately: a further 14 could not be assessed. Each report was graded inadequate overall.”^19^ Unfortunately, as Dal-Ré and colleagues concluded upon reviewing those results, “most universities and research institutions will continue handling alleged research misconduct cases with their own procedures, many not meeting reasonable standards and lacking transparency.”^20^

Our proposed checklist formed the basis of a 2019 checklist from the Association for the Promotion of Research Integrity in Japan^21^ and of a similar checklist by editors of major cardiothoracic journals.^22^ The journal editors, who like others sometimes receive reports, or their summaries, as part of communications with universities requesting corrections to the scientific record, noted they “have been hampered in dealing appropriately with some allegations of misconduct when institutional investigations have been poorly managed, have reached ambiguous or unsupported decisions or have conducted their investigation extremely slowly, often extending over several years.”^23^ The editors’ goal is to “help institutions provide reports that editors find helpful in making appropriate decisions about submitted or published manuscripts that may be seriously flawed.”^24^ We hope these approaches will lead to improved standards and outcomes.

Loikith and Bauchwitz called for improved regulations and audits for misconduct investigations.^25^ Titus and Kornfeld have argued for similar post hoc inquiries.^26^ Robert has called for investigation of research administrators — those who are responsible for institutional reports.^27^ We believe making misconduct reports available would be a key part of any such efforts. The availability of these documents in an online repository would further serve as a form of community peer review, either formal or informal, and would be in keeping with one of the main goals of the open science movement: promoting greater transparency in publicly funded research. One might imagine that a repository of reports available for such review would encourage institutions to be more fulsome in their disclosures.

## What journals can do

A first step would be for retraction notices — often the only public-facing available information about misconduct — to state the existence of an institutional investigation, which we have previously recommended.^28^ But Xu et al. found that “most retraction notices (73.7%) provided no information about institutional investigations that may have led to retractions.”^29^ A recent study in which one of us took part found a similar result.^30^

To be fair, journals may want to move to correct the record before an institutional investigation is complete, or even in some cases when they are unaware that such an investigation is being undertaken. Journals have long complained that many universities are reluctant to share what they think is relevant information, and efforts to improve communication — including recommendations that institutions allow journals to quote from investigation reports — are welcome.^31^ But in these cases, as Xu et al. argue, “published retraction notices should be updated to mention institutional investigations,”^32^ suggesting the Committee on Publication Ethics strengthen its recommendations and make them mandatory, and we agree.

As Xu and colleagues write, doing so “sends a clear message that various institutional stakeholders are doing their job, either independently or collaboratively, to uphold the integrity of research and publication norms. Such a message can deter potential offence and win back the public’s trust in the self-governance and self-correction of academic research.”^33^

## Who does confidentiality serve?

In the world of academic institutions, the principle of confidentiality is both critically important and frequently distorted. To be sure, a certain degree of caution is essential when institutions consider how much, if any, identifying information to disclose during their investigations and when in the process to do so. Protecting the privacy interests of the accused is not only a matter of policy but, for state agencies, at least, law — and for good reason: The public disclosure of damning but inaccurate information about a faculty member, for example, can have disastrous consequences for that person’s personal and professional standing.

And yet, the preservation of confidentiality is not in itself a societal good on par with, say, safeguarding the public trust in science, protecting the public health, or the effective stewardship of taxpayer resources.

The question of who benefits from reactionary rules about confidentiality is worth considering here. On its face, the answer would seem to be: researchers. But in fact, institutions have as much or even more to gain from asserting a strong right to anonymity during investigations into their faculty and staff. They have a powerful incentive to control the flow of information that might be damaging to their reputation, and to prevent outside parties — journalists, lawyers, potential funders, etc. — from conducting their own inquiries into a case of potential misconduct. Although that desire is valid as investigations are in process, and guilt or innocence has yet to be established, it loses potency once reports are complete. What is the rationale for keeping an investigation report confidential in the event a researcher at a public university has been found guilty of fraud? If the document redacts information, what is the explanation for doing so, and does that reason withstand scrutiny?

Evidence, albeit scant, indicates researchers who are transparent about their mistakes reap a “trust dividend” in the form of increased citations of their future publications.^34^ The reason for this bump, it would seem, is that other scientists may make the calculation that a peer who is open about problems with his or her output is likely to be more vigilant going forward.

We believe academic institutions would derive a similar benefit from greater transparency into their investigative processes, and to be seen living up to their own purported standards of rigor, openness, and honesty.

Disclosure of investigation reports serves another important purpose: namely, demonstrating to the public and relevant stakeholders that the inquiry addressed the proper scope and details of the case. Institutions often behave in ways that reflect motivated blindness, counterproductive processes, and misguided values and reactions to allegations of misconduct by their faculty and staff. As a result, the reports of such inquiries, while “official,” fail to inspire confidence that the truth was the goal. The case of Anil Potti, at Duke University, is one such example. Duke strongly discouraged, and in some instances ignored, whistleblowers, whose statements could have prevented patients from being exposed to Potti’s misconduct during clinical trials.^35^

## Constraints and how to mitigate or overcome them

Just as publishers have concerns about noting the existence of misconduct investigations in retraction notices, universities have noted legal and privacy concerns when refusing to release misconduct investigation reports. Universities in the United States often cite a “need to know” clause in CFR 42 part 93,^36^ which governs research misconduct, but that clause is not nearly as restrictive as some universities claim. The fact that reports are often subject to public disclosure laws in relevant jurisdictions demonstrates the porousness of the regulation.

A smaller concern voiced by universities and individual faculty is that investigation reports often include the names of the committee members, colleagues, witnesses to alleged misconduct, and others, which would subject them to risk and liability. That concern seems minimal given those accused of misconduct already know who the committee members were, as well as those interviewed in the inquiry. Redaction of the names of committee members would obviate this problem.

Similarly, universities express concerns about releasing reports of investigations that did not find misconduct, particularly if they include personnel-related allegations that may prejudice the public. While we believe it would be best to release the final reports of investigations regardless of the findings — just as publishing null and “negative” findings in the scientific literature is important to reflect reality — we can understand how some of these reports might be exempted from disclosure, but not by default. What those criteria should be is a topic worthy of more exploration and debate. We are planning a workshop in collaboration with the National Center for Principled and Research Ethics to consider these issues.

Similarly, we would support releasing only executive summaries of any initial inquiries that did not lead to full investigations, to limit the incentives for the small number of bad faith allegations made just to destroy a rival’s credibility.

To offer an imperfect analogy, the National Collegiate Athletic Association (better known as the NCAA) has created a set of community standards for its member institutions. Those schools that wish to participate in sanctioned events, such as the NCAA “March Madness” basketball tournament, have to follow those rules regardless of whether they are public or private. They may have different approaches to recruiting, educating, and otherwise treating their students, but they must all abide by the association’s regulations.

We believe a similar system could and should apply for the community of academic research. The best way to prevent idiosyncratic behavior — including cronyism, defensiveness, and opacity — is to create a framework for behavior against which exceptions can be judged to be either reasonable or warranted.

We agree with Siegerink et al., in their argument for a centralized jurisprudence platform for scientific misconduct, when they write, “Privacy is at odds with the completeness of data as well as the widely carried scientific notion of transparency.”^37^

The importance of transparency to the preservation of public trust in science should be presumed to outweigh the narrow aims of individual privacy protections.

Some skeptics of greater transparency for investigative reports argue that such publicity penalizes the unfairly accused — or even the fairly accused, depending on the severity of the accusations. But even in the criminal justice system, where the expectations for due process are clear and well-established, confidentiality ends at the moment a charge is entered into the court system. In other words, the charge becomes public at the very moment it becomes official.

A critical distinction between the legal system and academia is that a criminal charge immediately becomes public information. What’s more, those charges do not stem from allegations, but rather from a prosecutor, who has determined the evidence can support the case. Investigations of academic misconduct, on the other hand, frequently are triggered by the claims from anonymous whistleblowers or other individuals.

To be sure, some people are erroneously charged and ultimately convicted of crimes they did not commit, with significant consequences to their reputation and lives generally. However, the best way to mitigate the impact of these unfortunate cases is through more and not less publicity. As Louis Brandeis famously wrote, “sunlight is the best disinfectant.”

We feel the same is true in academia. Although of course those who commit misconduct are likely to suffer damage to their reputation, the fault is their own. On the other hand, those found innocent during an inquiry deserve to have that finding made clear to preserve their good name in the eyes of their peers and the public.

Some information in reports almost certainly will require redaction. What, for example, an individual witness said about a particular person is likely worth shielding. However, those details are procedural and surmountable with clear guidelines.

## The way forward

We recommend universities universally make investigation reports available as a default, with a clear explanation of any exemptions. We are not the first to make this plea. For example, our colleague Gunsalus did so in 2019: “We need systems that are as rigorous, open and accountable as the research that institutions seek to produce.”^38^ In 2022 De Peuter and Conix said universities “should publicly publish anonymized reports about the results of research integrity investigations, including sanctions and measures against whistleblower retaliation”^39^ in order to increase “potential whistle-blowers’ confidence in their institutions’ ability to safely, promptly, professionally, and satisfyingly investigate suspected breaches of integrity.”^40^

Notably, many, if not all, professional licensing boards make public their findings, including those from cases involving research misconduct that rises to the level of sanctionable. One counter to this observation may be that loss of licensure typically, although not always, results from criminal behavior, whereas most academic misconduct is not considered felonious. However, that argument misses the larger point: professional accreditation and good standing in the academic community (by which we mean any institution that accepts public funding, including but not limited to research dollars) imply fidelity to a standard of practice that is essential to continued membership. Failure to adhere to that standard can and should result in some degree of lustration.

The surge in public interest in scientific fraud that began in 2022 with news of an investigation into Stanford’s then-president Marc Tessier-Lavigne has not shown any significant signs of abating, particularly as stories about paper mills and other misbehavior make the front pages of newspapers around the world. That suggests politicians and others from outside of science may — as they have before — become understandably impatient with a lack of progress in combating misconduct and implement steps that scientists may find objectionable. Institutions should take steps toward transparency, including making the release of misconduct reports standard, if they are concerned about such moves.
